# Arrival of Chikungunya Virus in the New World: Prospects for Spread and Impact on Public Health

**DOI:** 10.1371/journal.pntd.0002921

**Published:** 2014-06-26

**Authors:** Scott C. Weaver

**Affiliations:** 1 Institute for Human Infections and Immunity, Galveston, Texas, United States of America; 2 Center for Tropical Diseases, Galveston, Texas, United States of America; 3 Department of Pathology, University of Texas Medical Branch, Galveston, Texas, United States of America

*For the first time in modern scientific history, chikungunya virus has established its mosquito-human transmission cycle in the Americas. The history of dengue control, recent findings on chikungunya strain variation, and public health preparedness indicate the likelihood of the further spread of this outbreak.*

**Figure 1:**
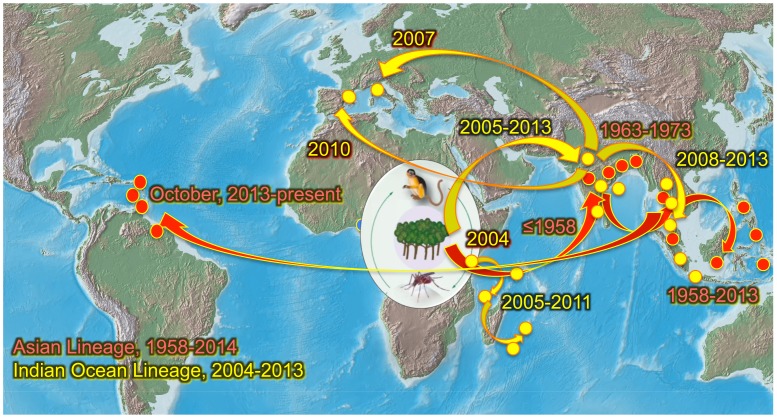
Map showing the distribution of chikungunya virus enzootic strains in Africa and the emergence and spread of the Asian lineage (red arrows and dots) and the Indian Ocean lineage (yellow arrows and dots) from Africa.

The mosquito-borne chikungunya virus (CHIKV; *Togaviridae*: *Alphavirus*) causes a febrile illness (chikungunya fever, or CHIK) typically accompanied by rash and severe, debilitating arthralgia. Pain and swelling are usually focused in the hands, wrists, ankles, and feet and can persist for years to cause not only major public health effects but also economic damage due to lost human productivity [1]. Most cases are not life threatening, although slightly increased mortality is associated with CHIKV infection. The virus is believed to have originated in Africa, where it still circulates enzootically among nonhuman primates, and is transmitted by arboreal *Aedes* mosquitoes (Figure 1) [2], [3]. These cycles lead to regular outbreaks of spillover infection in Africa, but most human cases result from CHIKV emergence into a human–mosquito cycle in urban areas of Africa, followed sometimes by spread beyond Africa. Evidence from historic accounts suggest that this emergence began as early as the 18th century in Indonesia and possibly the Americas, presumably via sailing ships that carried the essential ingredients for on-board circulation: susceptible humans and the peridomestic mosquito vector, *Aedes aegypti* [4]. Two other viruses that circulate in the same cycle, dengue and yellow fever, are also known to have caused outbreaks in port cities where this tropical mosquito was introduced, either temporarily during the summer in temperate climates where it cannot survive cold winters or permanently throughout tropical and subtropical regions of Asia, Europe, Australia, and the Americas.

Following its discovery in 1952, the first documented CHIKV emergence spread to generate urban outbreaks in India and Southeast Asia (Figure 1). This introduction has been traced to an Eastern/Central/Southern African (ECSA) enzootic CHIKV lineage that evolved sometime during or before the early 1950s [2], [3]. The resultant “Asian” endemic/epidemic CHIKV lineage persisted in Southeast Asia, where it continues to circulate sporadically in the urban cycle, transmitted among humans by *A. aegypti* without conclusive evidence of an enzootic component (Table 1). The second documented CHIKV emergence began in coastal Kenya in 2004 [5] and spread independently into islands in the Indian Ocean and to India, presumably via infected air travelers, a documented source of introductions [6]–[8]. Later, autochthonous transmission occurred in Italy [9] and France [10], initiated by infected travelers from India (Table 1). Although many imported cases were also detected in the Americas [6], including in dengue-endemic locations with both *A. aegypti* and *A. albopictus* vectors, no local transmission was detected. As with the Asian lineage, the etiologic CHIKV strain, called the Indian Ocean lineage (IOL) was again identified as a descendent from an enzootic ECSA strain [11]. However, some IOL adapted to a new vector, *A. albopictus*, through adaptive mutations in the E1 [12], [13] and E2 [14], [15] envelope glycoprotein genes. These mutations allowed the new epidemic IOL strains to use both *A. aegypti* and *A. albopictus* as vectors, resulting in millions of human cases. Because *A. albopictus* can survive cold winters and is generally less adapted to urban habitats than *A. aegypti*, IOL CHIKV strains adapted to this vector circulated both in temperate climates such as Italy [9] and in more rural habitats where the former species is more common than the latter [16].

**Table 1:** Representative chikungunya fever outbreaks documented in the literature.

Year	Location	Estimated number of cases	Virus genotype*	Notes	References
1952–1953	Tanzania	Incidence estimated at 23%	ECSA	Suspected vector *A. aegypti*	[26], [27]
1961–1963	Cambodia	Six confirmed	Not determined		[28]
1956, 1975–1977	South Africa	Not reported	ECSA	No *A. aegypti* involvement	[29]
1957, 1961–1962, 1971	Zimbabwe	38 suspected (one confirmed), 1962	ECSA	No *A. aegypti* involvement	[29]–[31]
1958, early 1960s	Thailand (Bangkok and other locations)	Estimated 40,000 cases in early 1960s	Asian	Suspected vector *A. aegypti*	[32]
1962–1965	India (various locations including Calcutta, Madras)	273 confirmed	Asian	Up to 38% human seroprevalence after outbreak, incidence in some locations estimated at 40%; principal vector *A. aegypti*	[33]–[36]
1962–1964	Bangkok, Thailand	44,000–72,000 estimated	Asian	Principal vector *A. aegypti*	[37]
1966	Viet Nam	Ten confirmed	Not determined	U.S. soldiers	[38]
1969	Nigeria	55 confirmed	Not determined		[39]
1998	Selangor State, Malaysia	51 cases reported	Not determined		[40]
1999–2000	Democratic Republic of Congo	40,000 estimated	ECSA		[41]
2004–2005	Coastal Kenya, Lamu Island	Not reported	IOL	Principal vector *A. aegypti* on Lamu Island	[5], [42], [43]
2005–2011	Comoros, Maurituis, La Reúnion	300,000 estimated in La Reúnion	IOL	Principal vector *A. albopictus* on La Reúnion	[11], [44], [45]
2005–2008	India, Sri Lanka	1.4–6.5 million	IOL (E1-226A or V in different outbreaks)	Vectors *A. albopictus* or *A. aegypti*, depending on location	[46], [47]
2006	Bagan Panchor, Malaysia	>200 reported	Asian		[48]
2006	Douala and Yaoundé, Cameroon	54 confirmed	ECSA	Suspected vector *A. africanus*	[49], [50]
2006–2007	Libreville, Gabon	Seven confirmed, 20,000 estimated	ECSA	Suspected vector *A. albopictus*	[51]–[53]
2007	Emilia Romagna, Italy	205 confirmed	IOL (introduced by a traveler from India)	Principal vector *A. albopictus*	[9]
2007–2008 (nonepidemic period)	Moshi, Tanzania	55 confirmed	Not determined		[54]
2008	Thailand	224 confirmed, 46,000 estimated	IOL	Suspected vector *A. albopictus*	[55], [56]
2008	Rural Malaysia	34 confirmed	IOL	Suspected vector *A. albopictus*	[57]
2008	Singapore	231 confirmed	IOL (E1-226A)	Principal vector *A. aegypti*	[58], [59]
2010	Fréjus, France	Two confirmed	IOL (E1-226A; imported from India)	Suspected vector *A. albopictus*	[10], [60]
2010	Ndangui, Gabon (forested region)	12 confirmed	Not determined	Principal vector *A. albopictus*	[61]
2010	Guangdong Province, China	173 suspected, ten confirmed	IOL	Suspected vector *A. albopictus*	[62]
2011	Cambodia	24 confirmed	IOL		[63]
2012	Bhutan	78 suspected	IOL (E1-226A)		[64]
2013–present	Caribbean Sea islands	>3,000 confirmed as of March 2014	Asian	Principal vector *A. aegypti*	[18]

*IOL strains had E1-226V unless otherwise noted.

During the ongoing IOL CHIK epidemics, the nearly completely naïve human populations in the Americas and the presence of both epidemic vectors, combined with the arrival of infected travelers, raised major concerns that an epidemic in the Caribbean and/or Latin America was inevitable [17]. However, with the gradual subsidence of epidemic transmission in many parts of Asia, this risk was perceived to have declined, because fewer infected travelers were documented in recent years.

Thus, the detection of active CHIKV circulation in Saint Martin beginning in October 2013 [18] was somewhat surprising. Furthermore, the characterization of the etiologic strain as belonging to the old Asian lineage rather than to the IOL was unexpected, considering that the former was viewed as displacing the latter in many parts of Asia [19]. However, because it apparently infects *A. aegypti* slightly more efficiently than CHIKV strains with the *A. albopictus*-adaptive E1 protein substitution [20], the Asian lineage may remain prevalent in urban areas of Asia, from which infected travelers are more likely to depart for global travel.

There is much bad news and only very limited good news in the 2013 CHIKV introduction into the Caribbean. The bad news includes: (1) CHIKV appears to be spreading nearly uncontrolled in the Caribbean, with over 4,300 confirmed cases as of May 23rd (Pan American Health Organization data). (2) Autochthonous transmission has resulted in at least 176 CHIK cases in French Guiana on the South American mainland. If transmission cannot be controlled quickly there, the historic inability to control dengue suggests that CHIKV will spread throughout Latin America. (3) Most of the Latin American population is presumably naïve, setting the stage for major epidemics and rapid spread. (4) Diagnostic capabilities for CHIKV in Latin America remain very limited, and it is possible that undetected circulation is already occurring in the region because of the difficulty in clinically distinguishing dengue from CHIK. And finally, (5) there could be the potential for CHIKV to establish an enzootic, monkey–human cycle in the Americas, as occurred for yellow fever virus hundreds of years ago after its importation from Africa [21].

If there is any good news related to this CHIK outbreak, it is that the etiologic strain, a member of the old Asian lineage, does not infect *A. albopictus* as efficiently as the adapted IOL strains, and is epistatically constrained in its ability to adapt to this vector via the E1-226 protein substitution [22]. This suggests that most CHIKV transmission in the Americas will occur via *A. aegypti*, which may limit geographic spread, particularly to temperate climates where this mosquito does not normally occur. However, *A. aegypti* reinfestation of most tropical and subtropical regions of Latin America since the 1970s [23], along with its persistence in the southern United States, leaves hundreds of millions of persons at risk for CHIKV infection. The presence of the closely related Mayaro alphavirus in South America could provide limited cross-protection [24], but this virus circulates enzootically, mainly in forested areas, where *A. aegypti*-borne CHIKV is expected to be less prevalent. Finally, the introduction of CHIKV during the beginning of the dry season in the Caribbean and northern hemisphere of Latin America may improve prospects for containing its spread, at least temporarily.

In summary, the prospects for controlling CHIKV circulation in Latin America since its arrival on the mainland of South America are not good, and many parts of the Americas are now at high risk for major epidemics. Because vaccines and specific antiviral therapies for CHIKV are not yet available [25], the only means for controlling its spread are reductions in *A. aegypti* populations and limiting human contact with this vector. It is therefore critical that public health officials implement robust surveillance based on existing dengue programs, establish local diagnostic capacity to test mosquitoes and patient sera from suspected cases, and develop outbreak response plans, including educational efforts to reduce contact with vectors. Health care workers should also be trained to include CHIK in their differential diagnoses for dengue-like illness and to optimally use available medications to alleviate the severe symptoms of CHIK.
